# Acute kidney injury in postoperative shock: is hyperoncotic albumin administration an unrecognized resuscitation risk factor?

**DOI:** 10.1186/s13741-018-0110-y

**Published:** 2018-12-14

**Authors:** Chiedozie I. Udeh, Jing You, Matthew R. Wanek, Jarrod Dalton, Belinda L. Udeh, Sevag Demirjian, Nadeem Rahman, J. Steven Hata

**Affiliations:** 1 Center for Critical Medicine, Anesthesiology Institute, 9500 Euclid Avenue, (J4-331), Cleveland, OH 44195 USA; 2 Department of Special Line Product Development, Progressive Insurance, 300 N. Commons Blvd, Cleveland, OH 44143 USA; 3 Department of Inpatient Pharmacy, Cleveland Clinic Pharm D, 9500 Euclid Avenue, Cleveland, OH 44195 USA; 4 Department of Quantitative Health Sciences, Lemer Research Institute, Cleveland Clinic, 9500 Euclid Avenue, Cleveland, OH 44195 USA; 5 Quality and Patient Safety Institute, NI-CORE, Neurological Institute, 9500 Euclid Avenue, Cleveland, OH 44195 USA; 6 Department of Nephrology, Glickman Urology and Kidney Institute, Cleveland Clinic, 9500 Euclid Avenue, Cleveland, OH 44195 USA; 7 Critical Care Institute, Cleveland Clinic, P.O. Box 112412, Al Maryah Island, Abu Dhabi, United Arab Emirates

**Keywords:** (National Library of Medicine-Medical Subject Headings): Albumin, Resuscitation, Acute kidney injury, Shock, Multiple organ failure

## Abstract

**Background:**

The use of hyperoncotic albumin (HA) for shock resuscitation is controversial given concerns about its cost, effectiveness, and potential for nephrotoxicity. We evaluated the association between early exposure to hyperoncotic albumin (within the first 48 h of onset of shock) and acute organ dysfunction in post-surgical patients with shock.

**Methods:**

This retrospective, cohort study included 11,512 perioperative patients with shock from 2009 to 2012. Shock was defined as requirement for vasopressors to maintain adequate mean arterial pressure and/or elevated lactate (> 2.2 mmol/L). Subsets of 3600 were selected after propensity score and exact matching on demographics, comorbidities, and treatment variables (> 30). There was a preponderance of cardiac surgery patients. Proportional odds logistic regression, multivariable logistic regression or Cox proportional hazard regression models measured association between hyperoncotic albumin and acute kidney injury (AKI), hepatic injury, ICU days, and mortality.

**Results:**

Hyperoncotic albumin-exposed patients showed greater risk of acute kidney injury compared to controls (OR 1.10, 95% CI 1.04, 1.17. *P* = 0.002), after adjusting for imbalanced co-variables. Within matched patients, 20.3%, 2.9%, and 4.4% of HA patients experienced KDIGO stages 1–3 AKI, versus 19.6%, 2.5%, and 3.0% of controls. There was no difference in hepatic injury *(*OR 1.16; 98.3% CI 0.85, 1.58); ICU days, (HR 1.05; 98.3% CI 1.00, 1.11); or mortality, (OR 0.88; 98.3% CI 0.64, 1.20).

**Conclusions:**

Early exposure to hyperoncotic albumin in postoperative shock appeared to be associated with acute kidney injury. There did not appear to be any association with hepatic injury, mortality, or ICU days. The clinical and economic implications of this finding warrant further investigation.

## Background

Resuscitation with administration of hyperoncotic albumin is controversial with regard to clinical outcomes and organ injury in shock. Hyperoncotic colloids exert higher osmotic pressure relative to human plasma, a characteristic exponentially and directly related to the colloid concentration. Thus, 5% albumin with a colloid osmotic pressure similar to plasma is isooncotic. Whereas 20% albumin with osmotic pressure nearly 8-fold higher than plasma is classified as hyperoncotic, as are hydroxyethyl starch solutions at 6% or greater (Hiippala et al. 1991; Tonnessen et al. 1993). Owing to this characteristic, hyperoncotic fluids have been used for resuscitation. Proponents contend that albumin could limit organ damage in shock states, by achieving hemodynamic goals with smaller fluid volumes and avoiding adverse consequences of hypervolemia.

The use of 25% albumin is partly based on the presumption that distribution of administered albumin is primarily intravascular. If so, albumin should raise colloid oncotic pressure and expand plasma volume in shock. However, evolving understanding of endothelial glycocalyx physiology has upended the classic Starling principles of trans-endothelial fluid and protein flux (Woodcock and Woodcock 2012; Chappell et al. 2008; Levick and Michel 2010; Becker et al. 2010). It is now established that the kinetics of albumin differ markedly between health and critical illness states; such that the normal trans-capillary escape rate of albumin increases 100–300% in shock states due to increased permeability (Nicholson et al. 2000). Consequently, the effectiveness of resuscitation with hyperoncotic albumin has been questioned. Clinically, this is underscored by the fact that when compared to less costly crystalloids, hyperoncotic albumin solutions have not conferred mortality or other outcome benefits in shock (Myburgh 2008; Roch et al. 2011; Polito and Martin 2013; Raghunathan et al. 2015; Schortgen et al. 2010). Indeed, outcomes and adverse effects of hyperoncotic albumin, other colloids, and crystalloids in acute resuscitation have been found to be similar (Finfer et al. 2004; Bunn and Trivedi 2012; Patel et al. 2014; Jiang et al. 2014; Stockwell et al. 1992a; Stockwell et al. 1992b).

Additionally, concerns have been raised periodically about this paucity of benefit, yet possible harm from hyperoncotic colloids, including potential nephrotoxicity and mortality (Brochard et al. 2010; Hartog et al. 2011; Perel et al. 2013; Zarychanski et al. 2013; Moran and Kapsner 1987). These culminated in systematic reviews and prompted consensus recommendations against continued use of hetastarches due to nephrotoxicity. As a result, hyperoncotic albumin may be the sole colloid in many hospitals (Brochard et al. 2010; Hartog et al. 2011; Perel et al. 2013; Zarychanski et al. 2013). However, other observational cohort studies of resuscitation have suggested increased risks of acute kidney injury (AKI) associated with hyperoncotic albumin in shock (Frenette et al. 2014; Schortgen et al. 2008). It is therefore crucial to clarify any renal impact of hyperoncotic albumin administration during the high-risk period of shock. Accordingly, the goal of this study was to evaluate the association between early exposure to hyperoncotic albumin (within the first 48 h of onset of shock) and acute organ dysfunction in post-surgical patients with shock.

## Methods

The Cleveland Clinic Institution Review Board approved this study and waived informed consent. The design was a retrospective, propensity score-matched cohort study with pre-specified analyses (Schuhlen 2014). The study sample included surgical patients (> 18 years) admitted to the surgical intensive care unit or the cardiovascular surgical intensive care unit at Cleveland Clinic from 2009 to 2012 with shock. Eligible patients were identified from institutional electronic medical records (Epic Systems Corporation©, Verona, WI, USA). Patients with missing data for renal function or vital status were excluded. Patient selection required an admission to the ICU with elevated serum lactate level > 2.2 mmol or administration of vasopressors within the first 12 h of ICU stay.

Consistent with recent consensus definitions for septic shock, shock was defined as requirement for vasopressors to maintain adequate mean arterial pressure and/or elevated lactate (Shankar-Hari et al. 2016). The cut-off threshold for lactate was 2.2 mol/L, reflecting the upper end of the reference range for serum lactate at the Cleveland Clinic clinical laboratory. In order to assess the impact of early exposure to hyperoncotic albumin, we identified patients who received 25% albumin during the first 48 h of resuscitation for shock. Flexbumin 25% ® (Baxter Healthcare Corp, Westlake Village, CA, USA), a 12.5 g, 50 ml solution, is the only formulation of hyperoncotic albumin used in the intensive care units at Cleveland Clinic.

The primary outcome was risk of acute kidney injury (AKI), defined by serum creatinine changes as per the Kidney Disease Improving Global Outcomes (KDIGO) criteria (Khwaja 2012). The KDIGO definition of acute kidney injury includes the following: stage 1: serum creatinine with a 1.5 to 1.9 times baseline or ≥ 0.3 mg/dl (≥ 26.5 μmol/l) increase; stage 2: serum creatinine 2.0 to 2.9 times baseline. Stage 3: serum creatinine of 3.0 times baseline or increase in serum creatinine to ≥ 4.0 mg/dL (≥ 353.6 μmol/L) or initiation of renal replacement therapy. Secondary outcomes included in-hospital mortality, duration of ICU stay, and risk of liver injury. Using a threshold for more severe dysfunction in the liver component of the SOFA score, hepatic injury was defined as total bilirubin > 6.0 mg/dl (Vincent et al. 1996).

### Statistical analysis

SAS software version 9.3 (SAS Institute, Cary, NC, USA) was used for statistical analysis. Sample size estimation was based on the CRYCO study which had overall incidence of acute renal events of 17% (Schortgen et al. 2008). In that study, acute renal events occurred in 10% and 35% of crystalloid and hyperoncotic albumin exposed patients, respectively. Similarly, renal injury after cardiac surgery has been reported with an incidence of 25% (Bastin et al. 2013). Based on these studies, assuming 20%, 5%, and 5% rates of stage 1–3 AKI respectively, 4000 subjects would be required for statistical power of 0.90 to detect an odds ratio of 0.8 for an ordinal outcome.

To control for confounders, propensity score and exact matching based on Elixhauser comorbidity measures, demographics, and treatment variables was performed to balance the albumin-exposed and non-exposed patients (Tables 1 and 2) (Elixhauser et al. 1998). During the period covered by our study, physiologic scoring indices for acuity were not consistently available in the study ICUs. Also at that time, there was not yet institutional or national consensus on the use of hetastarch, which approximately 50% of our patients received. Accordingly, the American Society of Anesthesiologists (ASA) physical status classification, and exposure to hetastarch were included as variables for matching the cohorts. Vasopressors were included as single variable for the development of the model, because the clinical data were insufficient to differentiate effects on AKI risk among vasopressor types (Group. KDIGOKAKIW 2012).

**Table 1:** Demographic and perioperative characteristics of albumin-exposed and non-exposed patients before and after matching with respect to absolute standardized difference

Variable	All patients			Matched patients		
	Albumin (*N* = 4669)	Control (*N* = 6843)	ASD^*^	Albumin (*N* = 3600)	Control (*N* = 3600)	ASD^*^
Age, years	67 ± 13	63 ± 15	0.238	66 ± 13	66 ± 13	0.015
Gender (male), no. (%)	2882 (62)	4203 (61)	0.006	2239 (62)	2269 (63)	0.017
Race			0.087			0.018
Caucasian	4197 (90)	6000 (88)		3220 (89)	3239 (90)	
African American	256 (5)	522 (8)		198 (6)	185 (5)	
Others	216 (5)	321 (5)		182 (5)	176 (5)	
Body mass index, kg/m^2^	28.4 ± 6.5	29.1 ± 7.0	0.108	28.6 ± 6.3	28.6 ± 6.3	0.014
ASA physical status, no. (%)			0.274			0.067
I	18 (0)	46 (1)		11 (0)	5 (0)	
II	79 (2)	271 (4)		51 (1)	45 (1)	
III	985 (21)	2083 (30)		755 (21)	672 (19)	
IV	3587 (77)	4443 (65)		2783 (77)	2878 (80)	
Emergent surgery, no. (%)	198 (4)	433 (6)	0.094	151 (4)	139 (4)	0.017
Type of surgery ^†^, no. (%)			–			0.000
Replacement of aortic valve	1318 (37.5)	1495 (37.3)		1120 (37.3)	1120 (37.3)	
CABG artery-vein single	750 (21.4)	714 (17.8)		621 (20.7)	621 (20.7)	
Replacement of mitral valve	420 (12.0)	448 (11.2)		391 (13.0)	391 (13.0)	
Repair of mitral valve	343 (9.8)	411 (10.3)		283 (9.4)	283 (9.4)	
Ascending aortic graft	287 (8.2)	234 (5.8)		225 (7.5)	225 (7.5)	
Revise ventricular muscle	108 (3.1)	201 (5.0)		96 (3.2)	96 (3.2)	
Transplantation of liver	75 (2.1)	192 (4.8)		73 (2.4)	73 (2.4)	
Exploration of abdomen	100 (2.9)	133 (3.3)		108 (3.6)	108 (3.6)	
Repair of mitral valve	84 (2.4)	74 (1.9)		61 (2.0)	61 (2.0)	
CABG arterial single	27 (0.8)	106 (2.6)		25 (0.8)	25 (0.8)	
Duration of surgery, (h)	7.0 ± 2.4	6.5 ± 2.6	0.198	6.9 ± 2.1	6.8 ± 2.1	0.042
Intraoperative time-weighted average of MAP, mmHg	75.4 ± 6.6	77.4 ± 8.3	0.269	75.3 ± 6.1	75.1 ± 6.3	0.006
Use of hydroxyethyl starch, no. (%)	2326 (50)	3524 (52)	0.034	1854 (52)	2088 (58)	0.131
Use of furosemide, no. (%)	224 (5)	179 (3)	0.116	112 (3)	87 (2)	0.042

*ASA* American Society of Anesthesiologist, *ASD* absolute standardized difference, *MAP* mean arterial blood pressure, *TWA* time-weighted average

^*^ASD absolute difference in means or proportions divided by the pooled standard deviation; imbalance was defined as an ASD greater than 0.046 (i.e., $1.96\times \sqrt{\frac{1}{3600}+\frac{1}{3600}}$)

^†^Ten most frequent procedures are listed

**Table 2:** Comorbidities of albumin-exposed and non-exposed patients before and after matching with respect to absolute standardized difference

Comorbidity, no. (%)	All patients			Matched patients		
	Albumin (*N* = 4669)	Control (*N* = 6843)	ASD^*^	Albumin (*N* = 3600)	Control (*N* = 3600)	ASD^*^
Congestive heart failure	1985 (43)	1888 (28)	0.317	1397 (39)	1440 (40)	0.024
Vascular disease	2509 (54)	2571 (38)	0.329	1924 (53)	1974 (55)	0.028
Pulmonary circulation disease	454 (10)	457 (7)	0.111	293 (8)	294 (8)	0.001
Peripheral vascular disease	970 (21)	1094 (16)	0.124	682 (19)	654 (18)	0.020
Hypertension, uncomplicated	2960 (63)	3689 (54)	0.194	2232 (62)	2264 (63)	0.018
Hypertension, complicated	41 (1)	91 (1)	0.043	35 (1)	41 (1)	0.017
Paralysis	42 (1)	87 (1)	0.036	30 (1)	28 (1)	0.006
Chronic pulmonary disease	780 (17)	910 (13)	0.096	548 (15)	555 (15)	0.006
Diabetes w/o chronic complications	881 (19)	1152 (17)	0.053	671 (19)	673 (19)	0.001
Diabetes w/ chronic complications	112 (2)	159 (2)	0.005	86 (2)	91 (3)	0.009
Hypothyroidism	590 (13)	691 (10)	0.080	429 (12)	410 (11)	0.017
Renal failure	517 (11)	615 (9)	0.069	374 (10)	362 (10)	0.011
Liver disease	165 (4)	283 (4)	0.032	137 (4)	118 (3)	0.029
Peptic ulcer disease (non-bleeding)	0 (0)	2 (0)	0.025	0 (0)	1 (0)	0.025
Lymphoma	130 (3)	193 (3)	0.002	104 (3)	102 (3)	0.004
Metastatic cancer	49 (1)	74 (1)	0.003	32 (1)	25 (1)	0.023
Solid tumor w/out metastasis	451 (10)	637 (9)	0.012	324 (9)	317 (9)	0.007
Rheumatoid arthritis/collagen vas	102 (2)	167 (2)	0.017	86 (2)	82 (2)	0.007
Coagulopathy	1784 (38)	1570 (23)	0.336	1192 (33)	1145 (32)	0.028
Obesity	234 (5)	341 (5)	0.001	169 (5)	186 (5)	0.022
Weight loss	581 (12)	620 (9)	0.109	373 (10)	356 (10)	0.016
Fluid and electrolyte disorders	3282 (70)	3873 (57)	0.287	2481 (69)	2546 (71)	0.039
Chronic blood loss anemia	59 (1)	106 (2)	0.025	51 (1)	50 (1)	0.003
Deficiency anemias	911 (20)	1184 (17)	0.057	685 (19)	686 (19)	0.001
Alcohol abuse	84 (2)	123 (2)	0.000	61 (2)	75 (2)	0.029
Drug abuse	27 (1)	58 (1)	0.032	22 (1)	16 (0)	0.024
Psychoses	159 (3)	277 (4)	0.034	133 (4)	129 (4)	0.006

^*^ASD absolute difference in means or proportions divided by the pooled standard deviation; imbalance was defined as an ASD greater than 0.046 (i.e., $1.96\times \sqrt{\frac{1}{3600}+\frac{1}{3600}}$)

We estimated each patient’s probability of receiving albumin (their propensity score) using logistic regression with albumin as the outcome, and using all pre-specified potential confounders, except for type of surgery. We then 1-to-1 matched albumin-exposed and control patients using a greedy distance matching algorithm (SAS macro: gmatch) (Division of Biomedical Statistics and Informatics, Mayo Clinic (HSR CodeXchange) 2003). Successful matches were restricted to patients with the same type of surgery (by CPT code) and those whose logit function of the estimated propensity scores (i.e.,$\log \left(\widehat{p}/\left(1-\widehat{p}\right)\right.$ estimated propensity score) were within 0.2 of the standard deviation of the logit of the propensity score (i.e., 0.2 × 0.63 = 0.126) of one another. Assessment of balance of the co-variables was performed using absolute standardized differences (ASD), i.e., difference in means or proportions divided by the pooled standard deviation). Imbalance was defined as an absolute standardized difference > 0.046 (1.96 ×  √ (1/3600 + 1/3600). Any imbalanced co-variables would be entered into the models comparing albumin and control patients on outcomes to reduce potential confounding.

We used proportional odds logistic regression model to assess the association between the exposure to albumin and AKI. This sort of model accounts for the ordinal nature of the response variable (i.e., “no AKI” better than “stage 1 AKI” better than “stage 2 AKI” better than “stage 3 AKI”). It yields an estimate of the relative odds of developing a more severe stage of AKI for albumin versus by control patients. These estimates are assumed to be proportional across the risk spectrum, i.e., the relative odds of AKI (of any stage) vs. no AKI are assumed to be equivalent to the relative odds of stage 2 or higher AKI vs. either no AKI or stage 1 AKI.

For the secondary outcomes, the cohorts were compared using multivariable logistic regression or Cox proportional hazard regression, as appropriate. We applied the Bonferroni correction to control for inflated type I error, thereby using a significance level of 0.017. The summary statistics were reported as mean ± standard deviation for normally distributed continuous variables or median [first-third quartiles] for non-normally distributed continuous variables or number (%) for categorical variables, as appropriate. The normality was assessed by Shapiro-Wilk test. The statistical significance level was 0.05.

## Results

**Fig. 1:**
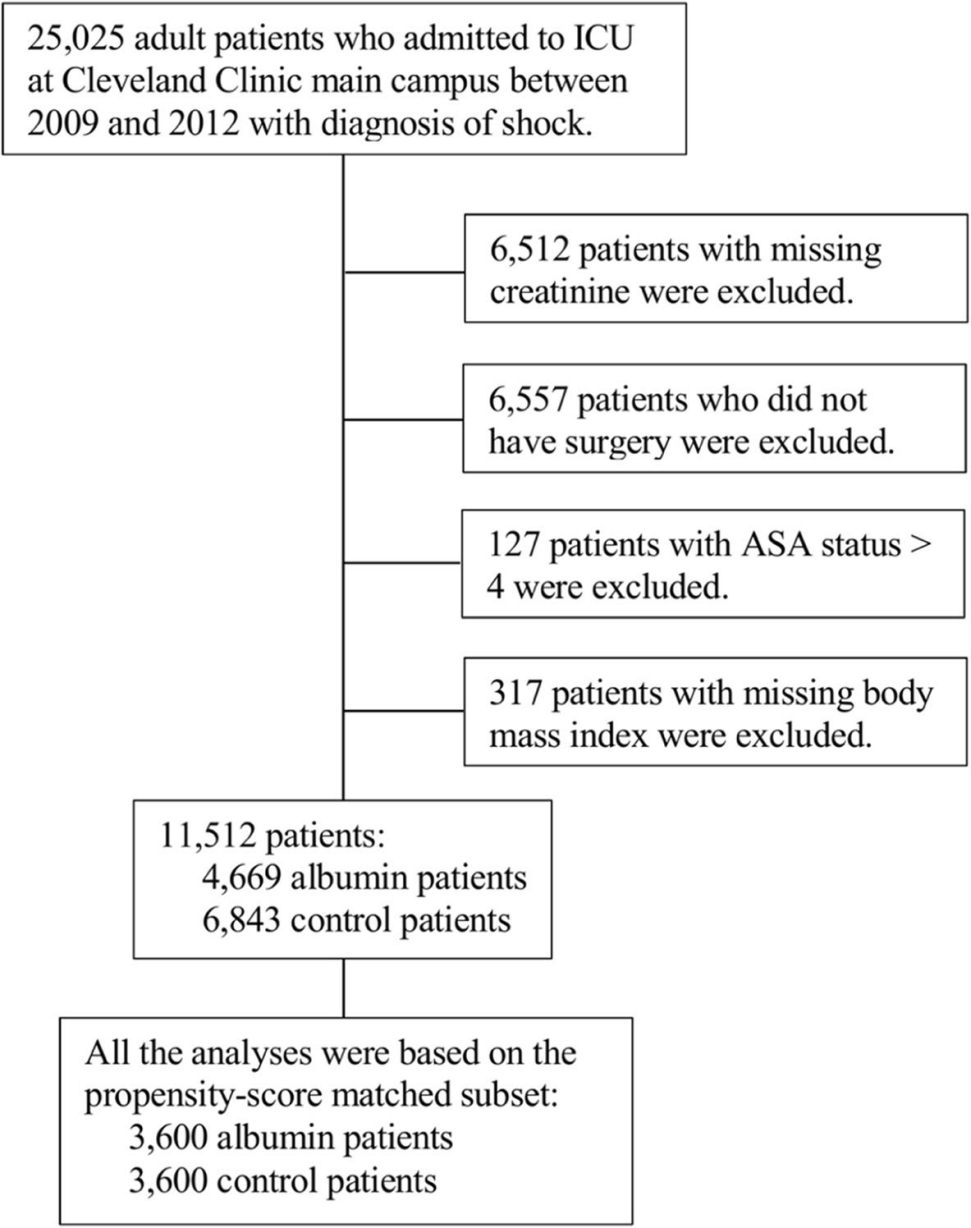
Patient selection flow chart

We identified 25,025 consecutive surgical ICU patients with shock during the study period. After excluding patients who had not undergone surgery and those with missing data, 11,512 patients were selected, 4699 of whom had received hyperoncotic albumin within the first 48 h of ICU admission. Covariate matching yielded 3600 hyperoncotic albumin-exposed patients successfully matched with 3600 control patients (Fig. 1). The demographic and perioperative clinical characteristics of the two groups are depicted in Tables 1 and 2, before and after matching. Most were Caucasian, 62% of male gender, with mean age of 66 ± 13 years, with a preponderance of cardiac surgical patients.

**Fig. 2:**
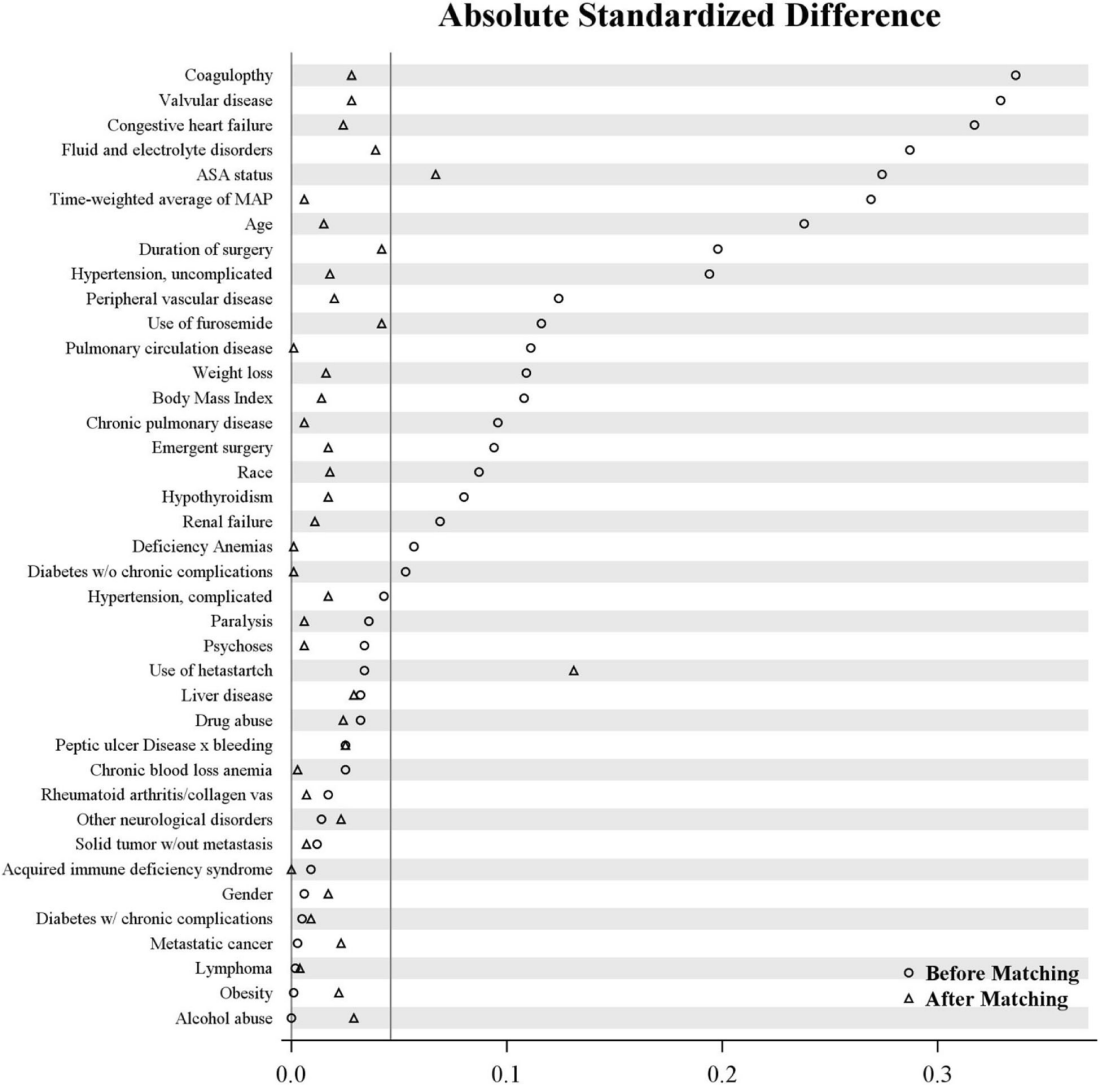
Absolute standardized difference of covariates used for propensity score matching based on albumin exposure. This plot depicts the absolute standardized difference (ASD) (*x* axis) of major clinical covariates (*y* axis) used to estimate the propensity score within the matched groups based on albumin exposure. The circles depict the ASD before matching, and triangles after matching

Propensity score techniques successfully balanced the variables in the matched subset (Fig. 2). Only ASA physical status and hetastarch exposure remained slightly imbalanced between the matched cohorts, using our ASD threshold of 0.046. (ASD 0.07 and 0.13, respectively). We therefore re-adjusted for these factors when comparing hyperoncotic albumin patients to their matched controls on the study outcomes.

**Fig. 3:**
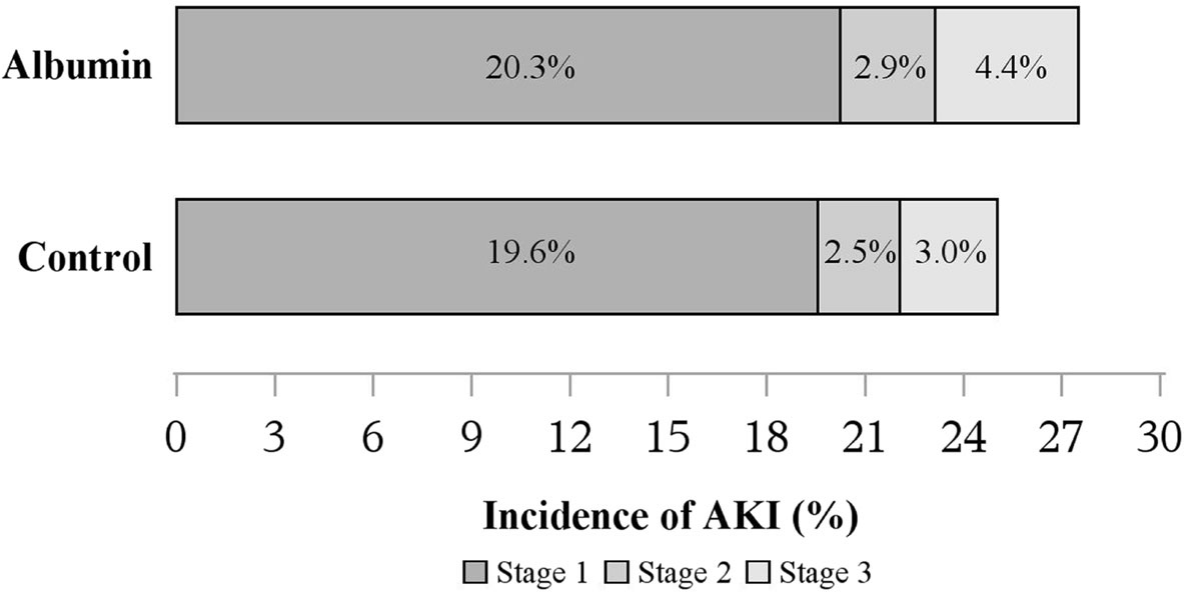
Incidence of KDIGO stages 1–3 acute kidney injury for matched albumin and control patients

Within the propensity-matched patients, 20.3%, 2.9%, and 4.4% of hyperoncotic albumin-exposed patients experienced stages 1, 2, and 3 of acute kidney injury, as compared to 19.6%, 2.5%, and 3.0% of control patients (Fig. 3). Patients exposed to hyperoncotic albumin were 10% more likely (OR 1.10, 95% CI 1.04, 1.17) to have been assigned a more severe AKI classification than their matched controls (*P* = 0.002), after adjusting for the imbalanced co-variables (Table 3).

**Table 3:** Associations between hyperoncotic albumin exposure and outcomes among the propensity score matched patients

Primary outcome	Albumin *N* = 3600	Control *N* = 3600	Odds ratio (95% CI) (albumin/control)	*P* value
Acute kidney injury			1.10 (1.04, 1.17)	0.002
No injury	2610 (72.5)	2699 (75.0)		
Stage 1 injury	729 (20.3)	704 (19.6)		
Stage 2 injury	104 (2.9)	90 (2.5)		
Stage 3 injury	157 (4.4)	107 (3.0)		
Secondary outcomes			Odds ratio (98.3% CI)^†^	
Liver injury	137 (3.8)	118 (3.3)	1.16 (0.85, 1.58)	0.25
In-hospital mortality	114 (3.2)	127 (3.5)	0.88 (0.64, 1.20)	0.32
			Hazard ratio (98.3% CI) ^†^	
Length of ICU stay^‡^ (h)	64 [41, 119]	50 [29, 115]	0.95 (0.90, 1.00)	0.03

^†^*P* value < 0.017 was considered significant (i.e., 0.05/3 = 0.017, Bonferroni correction)

^‡^Discharges for those patients who died in hospital were considered as non-events and censored at the longest observed length of stay

In contrast, no statistically significant differences were found for any of the secondary outcome measures. The estimated odds ratio for liver injury was 1.16 (98.3% CI 0.85, 1.58) (hyperoncotic albumin versus control, *P* = 0.25). Likewise, in-hospital mortality between hyperoncotic albumin exposed and non-exposed patients was not significantly different (OR 0.88, 95% CI 0.64, 1.20) (*P* = 0.32) (Table 3).

**Fig. 4:**
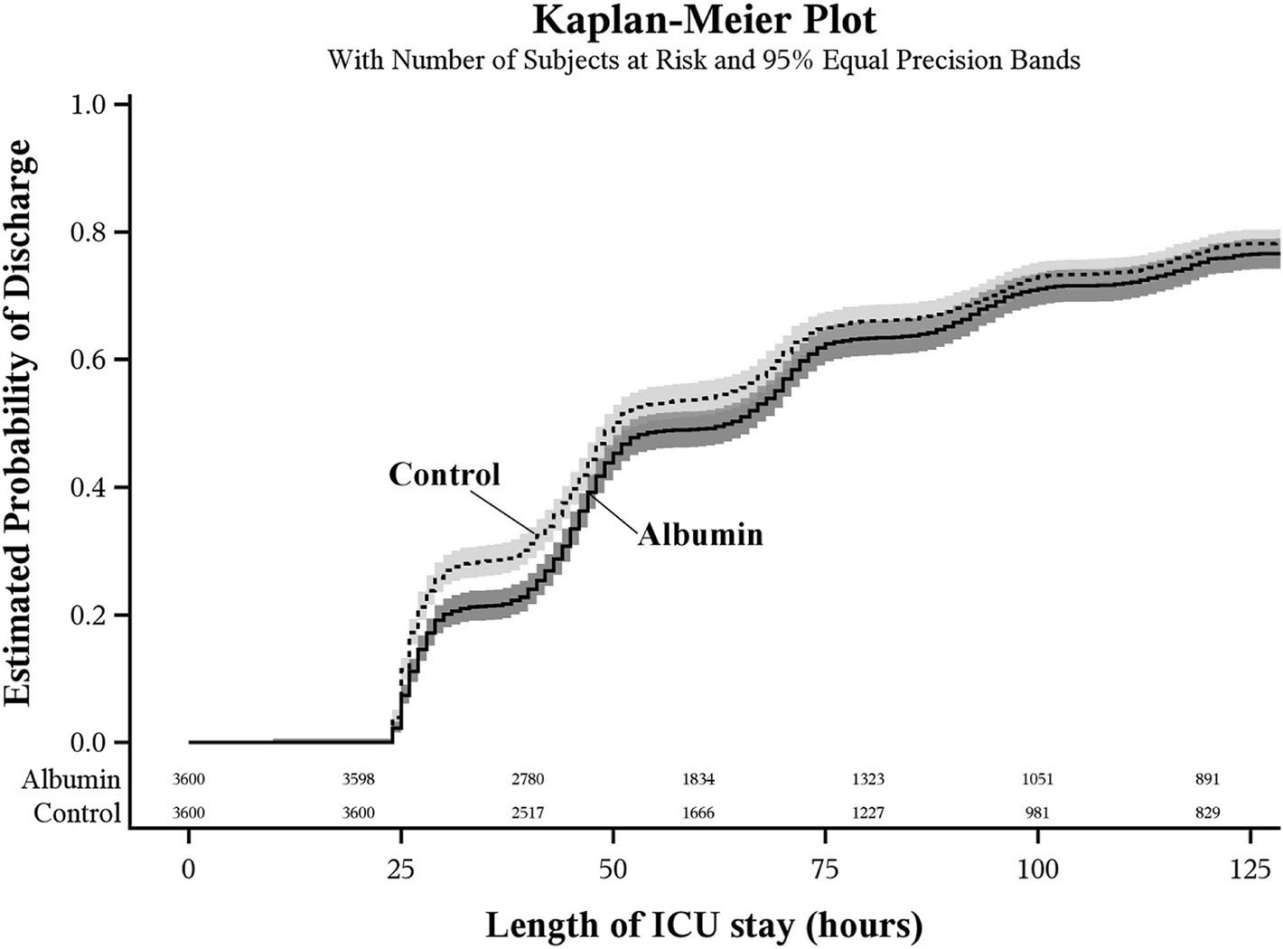
Kaplan-Meier plot showing duration of ICU stay for propensity score matched albumin and control patients

The estimated median duration of ICU stay was 64 h [interquartile range 41 to 119] for albumin patients and 50 h [interquartile range 29 to 115] for controls (Table 3). We did not observe any difference in length of ICU stay; log-rank test *P* = 0.02 > the Bonferroni corrected significance criterion of 0.017. The estimated hazard ratio for length of ICU stay was 1.05 (98.3% CI 1.00, 1.11) for hyperoncotic albumin vs. control patients, after adjusting for the imbalanced co-variables (*P* = 0.03) (Table 2). Discharges for patients who died in the ICU were considered as non-events and censored at the longest observed length of stay (Fig. 4).

## Discussion

Our results suggest that early administration of 25% albumin in postoperative shock may be associated with a more severe degree of AKI. Studies have indicated that development of AKI is associated with increased morbidity and mortality. For instance, 30–50% of septic patients develop AKI and in the sub-group that requires dialysis, 90-day mortality can be as high as 50% (Honore et al. 2015). Other investigators have previously challenged the perception of benefit, and/or low risk, of hyperoncotic albumin in the critically ill (Schortgen et al. 2008; Geer et al. 1976; Goodwin et al. 1981; Grootendorst et al. 1988). Raghunathan et al. conducted a multi-center retrospective cohort analysis of over 60,000 medical patients with septic shock (Raghunathan et al. 2015). They found that co-administration of colloids (including albumin) with crystalloids during initial resuscitation was associated with increased costs and length of stay, yet without improved survival. Notably, after matching our subjects, significantly more patients had received hetastarches in the control group. While we adjusted for this difference in the analysis of outcomes, the increased incidence of AKI found in the albumin-exposed group of our study was contrary to what may be expected considering hetastarch exposure and supports our findings.

Earlier in 2008, Schortgen et al. reported an increased risk of AKI in the CRYCO study, a multicenter, prospective observational cohort study of patients with shock (Schortgen et al. 2008). They reported an overall incidence of a renal event of 17.4%, defined as a 2-fold increase in serum creatinine or need for dialysis. Among those exposed to hyperoncotic albumin, there was a significant risk of AKI [OR 5.99, 95% CI 2.75–13.08] and ICU mortality [OR 2.79, 95% CI 1.42—5.47]. Similar to our results, they found no increase in hepatic injury [OR 1.04, 95% CI 0.58–1.89]. The smaller effect size notwithstanding, the increased risk of AKI identified in our study is concordant with the CRYCO study.

In contrast, the ALBIOS study of targeted albumin replacement in sepsis found neither nephrotoxicity nor mortality difference with albumin replacement (Caironi et al. 2014). This reinforces the concept of hypo-albuminemia as a consequence and not a cause of critical illness (Fleck et al. 1985; Hubner et al. 2016).

In 2016, Frenette et al. reported a 2-fold increased risk of AKI in cardiac surgery patients who received albumin (5% and 25%) (Frenette et al. 2014). Supporting a hypothesis of causation, they also found that the risk of AKI was dose-dependent. However, Lee et al. reported a 50% decrease in AKI in cardiac surgery patients treated with 20% albumin preoperatively for serum albumin levels less than 4 g/dl (Lee et al. 2016). Major surgery—like sepsis—is strongly associated with denudation of the endothelial glycocalyx (Steppan et al. 2011). Thus, Lee’s study suggests that timing of administration, and the clinical context might modulate the effects of hyperoncotic albumin. Notably, their control group received albumin postoperatively (both arms eventually receiving similar total amounts), which may explain the magnitude of the observed effect. Ordinarily, cardiac surgery has a relatively high incidence of AKI, attributable to the perioperative physiologic perturbations from bleeding, cardiopulmonary bypass, and the associated systemic inflammatory response which could presage the occurrence of AKI (Bastin et al. 2013). Accordingly, the preponderance of cardiac surgical patients in our cohort, a post hoc finding, raises concern that early exposure to hyperoncotic albumin in such patients may exacerbate the risk, leading to a more severe stage of AKI.

It is not yet known what might explain such paradoxical harm from hyperoncotic albumin. Albumin is a structural component of the intact endothelial glycocalyx and is a plasma anti-oxidant (Becker et al. 2010). In vitro, albumin inhibits apoptosis of endothelial cells if the intercellular adhesions are intact (Leitch et al. 2013; Zoellner et al. 1996). Therefore, it has been postulated, but not yet clinically demonstrated, that albumin supplementation in shock may help restore the glycocalyx (Nicholson et al. 2000; Leitch et al. 2013; Zoellner et al. 1996). Perhaps albumin administration prior to the onset of endothelial glycocalyx injury may be protective but further study is needed before recommending such preemptive resuscitation. However, as experience with other human blood products shows, the safety or beneficence of albumin cannot be assumed without question. Indeed, no two lots of human albumin solutions are identical, even from the same manufacturer. The solutions include molecules with a range of post-translational modifications that may attenuate their chelation or anti-oxidant capacity (Leitch et al. 2013). As such, any effects of albumin in shock are conditional on many as yet undetermined factors.

The pathophysiology of AKI after colloid exposure has been attributed to altered plasma oncotic forces and osmotic nephrosis described with hetastarches (Cittanova et al. 1996; Blasco et al. 2008; Schortgen et al. 2001). Basic research has proposed mechanisms that could promote renal injury as albumin filtered through glomeruli can promote renal interstitial inflammation, and dose-dependent increases in pro-inflammatory gene expression, TNF-alpha levels, and NF-kappa beta activity (Nicholson et al. 2000; Drumm et al. 2001a; Wheeler et al. 2011; Drumm et al. 2002; Drumm et al. 2001b; Gioannini et al. 2002; Kremer et al. 2011; Neuhaus et al. 2012). Another possible mechanism could be the adverse effect of albumin infusions on intracellular fluid volume demonstrated by Ernest et al. in both septic and postoperative cardiac surgery patients (Ernest et al. 1999; Ernest et al. 2001). They found that compared to saline, 5% albumin increased extracellular fluid volume, expanding both the plasma and interstitial compartments beyond the infused volume; thus, leading to the inference that the extra volume was derived from the intracellular space. Extrapolating from this in the setting of shock-induced hyper-permeability with overall plasma volume loss, hyperoncotic albumin may indeed raise plasma volume but at the cost of further intracellular dehydration—potentially stressing an intracellular milieu already deranged by shock. Any benefit from plasma volume expansion may thus be countered by intracellular dehydration.

With respect to secondary outcomes, our investigation did not support association of hyperoncotic albumin with increased risk of liver injury, in-hospital mortality, or ICU length of stay. This was concordant with the study by Raghunathan et al., which found an inconsistent association between colloid exposure and mortality (Raghunathan et al. 2015). Our findings though differ from the CRYCO study, which showed a significant increase in risk of mortality associated with hyperoncotic albumin without risk of liver dysfunction after covariate adjustment (Schortgen et al. 2008).

The strengths of our study include evaluating effect of 25% albumin exposure early during the course of shock, addressing the period of greatest hemodynamic instability, and potential for renal injury. We leveraged a large high-risk patient population and electronic medical records of our institution. To limit bias and misclassification risk, we analyzed consecutive patients, identified using objective criteria. Also, diagnostic codes for the clinical covariates were applied by professionals blinded to the study. The analysis used propensity matching of covariates to limit differences in severity of illness among the patient samples. Moreover, we set a threshold for assessing imbalance after propensity score matching by using an absolute standardized difference of < 0.05 between the groups. This is far stricter than the conventional threshold of 0.2.

Our study has inherent limitations because it is a single center, retrospective study which limits generalizability. Although we matched for potential confounders, the role of other clinical factors, not retrievable from our institution’s database, cannot be excluded as effect modifiers or confounders. We could not quantify the dose of 25% albumin, or characterize the type, and dose of crystalloids administered (0.9% saline or lactated Ringer’s solution). This precluded assessment of dose-dependency with outcomes. This was also the case with hetastarches; however, our institutional protocols for hetastarches then, limited their use to no more than 1 L to avoid coagulopathy.

Confounding by indication can affect validity of retrospective, observational research of comparative effectiveness, and safety (Psaty and Siscovick 2010). To limit the risk of residual confounding, we adjusted for multiple factors, potentially associated with the primary outcome of AKI, and controlled for exposure to hetastarches.

Moreover, we note the concordance of our results with those of Raghunathan et al. (whose study specifically addressed confounding by indication by also using inverse probability weighting analyses for risk adjustment, yet found no benefit from the use of albumin). Furthermore, our approach using “big data” from an electronic medical record has been similarly used by other investigations to assess renal failure after kidney transplantation (Srinivas et al. 2017). These investigators showed that the use of large patient dataset significantly enhanced the predictive analytical models of graft function survival. Analysis of large datasets, may detect previously unsuspected risk factors for organ injury associated with established medications or management protocols.

The findings of this study are germane in supporting an association between hyperoncotic albumin exposure and AKI in early perioperative shock. Given the nephrotoxicity risks of hetastarches, the concordance of our findings with similar studies of resuscitation in shock supports a re-examination of the therapeutic indications of hyperoncotic albumin.

## Conclusions

The use of hyperoncotic albumin for acute resuscitation in shock is controversial given its economic costs, absence of beneficial clinical outcomes relative to crystalloids, and the potential for nephrotoxicity. Our study suggests that exposure to hyperoncotic albumin during the first 48 h of perioperative shock may be associated with an increased risk of acute kidney injury, particularly among post-cardiac surgery patients. Within the matched groups, we did not detect differences in risk of liver injury, ICU length of stay or in-hospital mortality. Given the retrospective nature of the study, we view our findings as preliminary, and indicative of a need for prospective, randomized studies to achieve greater validity, and to identify the mechanisms for this apparent toxicity.

## Abbreviations

ASD: Absolute standardized difference
CI: Confidence interval
CRYCO: Crystalloids or Colloids study
ICU: Intensive care unit
KDIGO: Kidney Disease Improving Global Outcomes
OR: Odds ratio
